# Awake Craniotomy for the Treatment of a Cortical Pseudoaneurysm in a Pregnant Patient

**DOI:** 10.7759/cureus.1921

**Published:** 2017-12-07

**Authors:** Hirad Hedayat, Daniel R Felbaum, John E Reynolds, Rashid M Janjua

**Affiliations:** 1 Neurosurgery, Global Neurosciences Institute; 2 Neurosurgery, Medstar Georgetown University Hospital; 3 Anesthesiology, Wake Forest University Baptist Medical Center; 4 Neurosurgery, Novant Health

**Keywords:** pseudoaneurysm, gun shot wound, surgery and pregnancy, anesthesia and pregnancy

## Abstract

Neurosurgical pathologies presenting during pregnancy are uncommon. If present, the situation creates a unique diagnostic, observational, and therapeutic challenge as both lives are placed at potential risk. Surgical procedures during pregnancy are approached carefully as physiological stressors associated with surgery and anesthesia may cause fetal or maternal compromise. We present the only known case of a pseudoaneurysm treated with an awake craniotomy, allowing us to abate the risks associated with general anesthesia in pregnancy.

A female suffered a superficially penetrating gunshot wound to the head for which she underwent a craniotomy with complete neurological recovery. She had complaints of intermittent headaches, dizziness, and tingling of her hands five months thereafter. The cerebral angiogram demonstrated an 8 mm pseudoaneurysm under her craniotomy site. A surgical repair of this aneurysm was undertaken in the 23rd week of pregnancy via an awake craniotomy with regional scalp block. The aneurysm was resected without complication, and the patient tolerated the procedure without neurological deficit during or subsequent to the operation.

Cerebrovascular pathology in pregnant patients remains a difficult situation that poses challenges associated with the pathology itself as well as the anesthetic implications inherent with operative management. The neurosurgical literature demonstrates that surgical management of cerebrovascular pathology is well-tolerated in pregnancy, and our case further demonstrates the capability of utilizing an awake craniotomy for the treatment of this type of lesion without causing a residual deficit.

## Introduction

Neurosurgical pathologies presenting during pregnancy are uncommon and create unique diagnostic, observational, and therapeutic challenges as both lives are placed at potential risk. Reports of cerebrovascular pathologies in pregnancy include intracranial hemorrhage due to arteriovenous malformations (AVMs), aneurysms, or eclampsia [1]. Other reported lesions include tumors, hydrocephalus, and traumatic pathologies [2].

Surgical procedures during pregnancy are approached carefully as physiological stressors associated with surgery and anesthesia may cause fetal or maternal compromise. There is a 9% reported incidence of premature labor associated with operations during pregnancy. Maternal safety concerns relate to the physiological adaptations associated with pregnancy and require modifications in standard anesthetic techniques. Fetal concerns relate to medication-induced teratogenicity and the avoidance of fetal asphyxia, hypovolemia, and preterm labor, particularly in the first trimester [1-2].

In a review of keywords on a MEDLINE database, we present the only known case of a pseudoaneurysm treated with an awake craniotomy allowing the risks associated with general anesthesia in pregnancy to be abated.

## Case presentation

**Figure 1:**
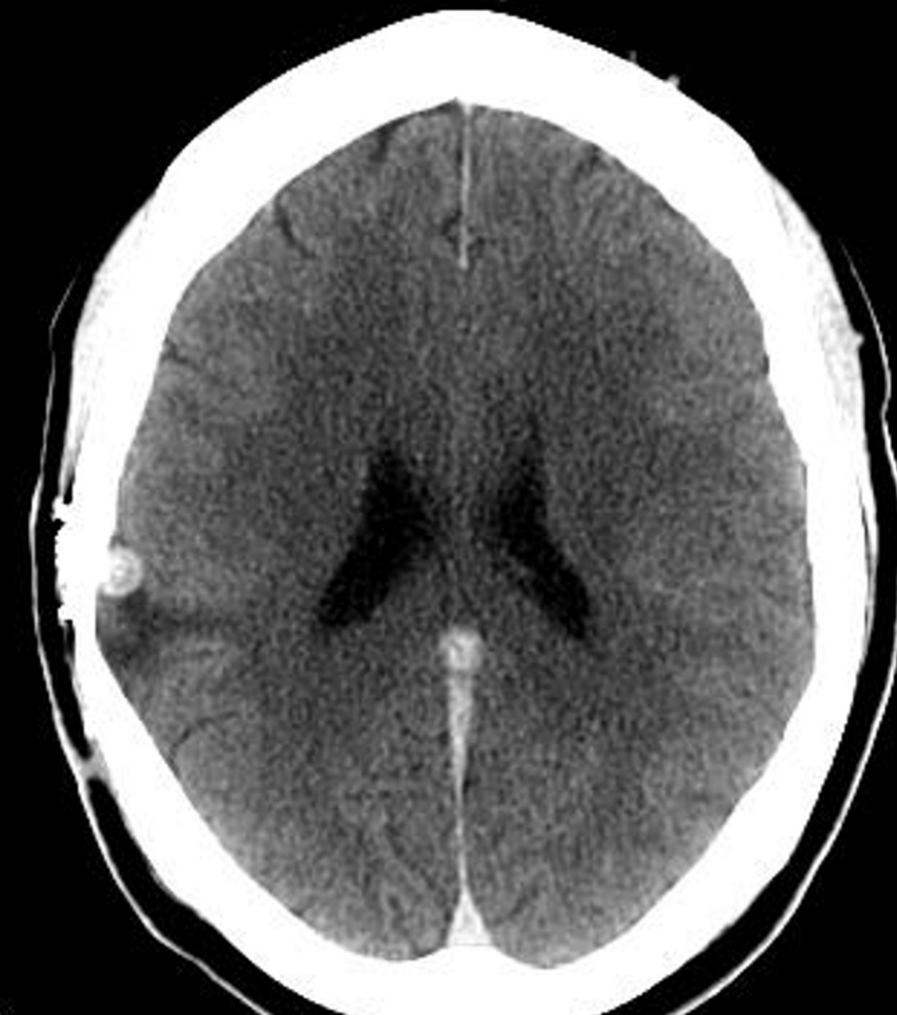
Computed tomography (CT) scan of head Computed tomography (CT) scan of head without contrast depicting hyperdensity deeper to the prior craniotomy site.

A female suffered a superficially penetrating gunshot wound to the head for which she underwent a craniotomy for debridement with dural and bony repair. She made a complete neurological recovery. Subsequently, she had complaints of intermittent headaches, dizziness, and tingling of her hands. Imaging of the brain with computed tomography (CT) suggested the presence of a 5 mm cortical pseudoaneurysm of the distal right middle cerebral artery (Figure 1).

**Figure 2:**
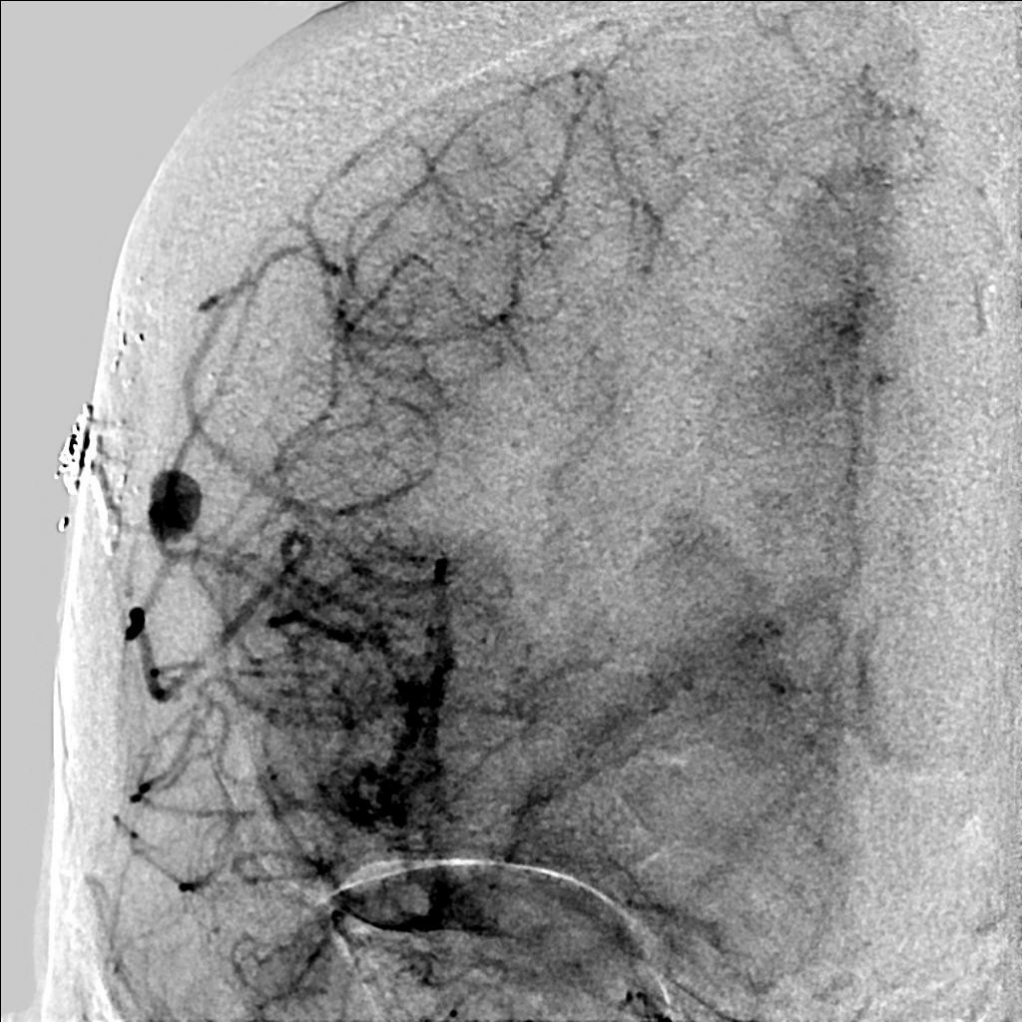
Cerebral angiogram Anteroposterior projection (AP) of right internal carotid artery revealing a cortical pseudoaneurysm.

The patient underwent a cerebral angiogram which confirmed an 8 mm pseudoaneurysm under her craniotomy site (Figure 2).

A surgical repair of this aneurysm was recommended. Upon pre-operative workup, she was noted to have an elevated β-HCG consistent with the second week of gestation. The patient was subsequently lost to neurosurgical follow-up. At the 22nd week of gestation, her obstetrician urged her to seek treatment for the aneurysm, suggesting that it was the optimal time for the patient and fetus. After coordinating the procedure between obstetrics, neonatology, and neuroanesthesia, the patient was taken to the operating room in her 23rd week of pregnancy. A regional scalp block was performed in addition to conscious sedation, and the original craniotomy incision and site was reopened using weight-appropriate dosing of local anesthetic without epinephrine, to avoid an inadvertent blood pressure elevation.

The craniotomy was extended to allow dural dissection on the underside of the prior craniotomy flap where the pseudoaneurysm was adherent. The pseudoaneurysm was dissected and primary reconstruction of the vessel attempted. This was unsuccessful in maintaining patency of the parent vessel. Aneurysmorrhaphy was attempted after temporary occlusion and this was unsuccessful as well. Temporary clips were replaced and the aneurysm was then excised and an end-to-end bypass performed. After several attempts, the patency of the bypass could not be maintained and it was sacrificed. Throughout this period, the patient’s neurological examination remained unchanged via frequent formal bedside assessments. Pre- and post-operative fetal heart monitoring demonstrated no abnormalities or variability. The patient subsequently delivered at term without neurological or obstetrical complications.

## Discussion

The challenge of cerebrovascular pathology in the pregnant patient demands the clinician adhere to strict neurosurgical standards while also caring for the obstetrical needs of the fetus. Notwithstanding, neurosurgical procedures during pregnancy may be associated with low maternal and fetal morbidity as well as mortality [2].

The overall incidence of cerebrovascular pathology is rare in pregnancy. Reported rates vary between 0.01-0.05% of all pregnancies and are the same as that of age-matched controls [3-7]. The overall incidence of intracerebral hemorrhage in the United States is 10-15 per 100,000 population per year. While rare in pregnancy, it accounts for 10% of all maternal deaths during pregnancy [7-8]. Intracerebral hemorrhage may be secondary to an AVM, which has a global prevalence of 0.04-0.52%. The overall maternal mortality rate associated with AVM-related hemorrhage is estimated at 28% [1]. The risk of recurrent hemorrhage during pregnancy from an untreated AVM or aneurysm has been estimated at 33-50% with an associated 50-68% maternal mortality rate [9]. The prevalence of cerebral aneurysms in the general population is dependent on the mode of data acquisition. Thus, reported as 0.4-6% in the general populace, it is estimated that the prevalence in the pregnant population is 1.8% [8]. The incidence of subarachnoid hemorrhage in pregnant women is reported to be five times higher than that of non-pregnant women. A recent analysis has estimated this risk as 0.05% to 1.4%, which is comparable to the risk of aneurysm rupture in the general population. Rupture of a cerebral aneurysm is associated with a fetal case-fatality of approximately 17% and maternal mortality of 35%; thus, aneurysmal ruptured accounts for 5-12% of all maternal death during pregnancy. It has been suggested that there is an increased tendency for aneurysmal subarachnoid hemorrhage with advancing gestational age due to increases in plasma volume and rates of pregnancy-induced hypertension [9]. Based on this data, surgical treatment of cerebrovascular pathology during pregnancy has been advocated based on neurosurgical principles [2].

Our case presents unique challenges and opportunities. In collaboration with Maternal-Fetal Medicine, it was felt most appropriate to proceed with surgery during the 18-23 week period of pregnancy as fetal heart rate (FHR) monitoring is feasible during this period and before FHR variability occurs at 25 weeks. This monitoring was performed in our patient pre-, intra-, and post-operatively.

The cortical location of the pseudoaneurysm afforded the ability to forego general anesthesia and allow for an awake craniotomy. Awake craniotomies are may begin with deep or unconscious sedation with or without airway control, although patient reassurance is of utmost importance. This approach has to be modified for the pregnant patient because they are considered to have a full stomach. Thus, pregnant patients are at a higher risk for aspiration of gastric contents. Hence, a pregnant patient needs an all-or-none approach: general anesthesia with a rapid sequence induction and a cuffed endotracheal tube or very light, conversant conscious sedation to assure airway protection should nausea and vomiting occur. We chose the latter, hence the scalp blocks to further diminish sedation requirements. The patient remained awake and conversant throughout the procedure, supported with intermittent delivery of fentanyl and diprovan, with great care to avoid hypercapnea. Further prophylaxis against aspiration included ranitidine and oral sodium citrate to decrease the acidity of gastric contents. Metoclopramide was used to improve gastric emptying. Dexamethasone and ondansetron were used to provide antiemetic effects. Likewise, in avoiding general anesthesia and allowing the patient to maintain her own respiratory rate, we avoided hyperventilation which may adversely affect the fetus by reducing cardiac output via diminished venous return. The ensuing hypocapnia produced uterine and umbilical artery vasoconstriction and the respiratory alkalosis caused a leftward shift of the oxygen-hemoglobin dissociation curve which reduced oxygen delivery to the fetus. Mannitol use was also avoided as it crosses the placenta and may cause fetal hypovolemia and dehydration, although use in an awake patient may be less tangible [10].

Amongst the physiological changes associated with pregnancy is a hypercoagulable state. This results in increases in most of the coagulation factors, enhanced platelet turnover, and increased clotting and fibrinolysis. Pregnancy is a state of compensated intravascular coagulation which may have contributed to the repeated intravascular thrombosis during surgery. Thus, at the risk of allowing a possible cortical hemorrhage at the bypass site secondary to enhanced fibrinolysis, the vessel was sacrificed. Administration of aspirin was considered intraoperatively but decided against due to a potential increased risk of fetal vascular disruptions. Despite the thrombosis of the cortical vessel bypass and subsequent sacrifice, the patient remained neurologically unchanged throughout the operation as well as in all her post-operative follow-up visits.

## Conclusions

Cerebrovascular pathology in pregnant patients presents a challenging treatment dilemma associated with the pathology itself as well as the anesthetic implications inherent with operative management. Our case of a traumatic cortical pseudoaneurysm is the only reported case of a cerebrovascular pathology in pregnancy treated with an awake craniotomy harnessing local anesthesia. The neurosurgical literature demonstrates that surgical management of cerebrovascular pathology is well-tolerated in pregnancy and our case further demonstrates the capability of utilizing an awake craniotomy for the treatment of this type of lesion without causing a residual deficit.
